# Design and development of an mHealth application for pressure ulcer care and caregiver support

**DOI:** 10.3389/fdgth.2025.1694486

**Published:** 2025-12-08

**Authors:** Shreenidhi Jogi, Vishal Shanbhag, Lakshay Chauhan, Siddhartha Chhauda, Utkarsh Dubey, Ajitha K. B. Shenoy, Elsa Sanatombi Devi

**Affiliations:** 1 Department of Medical-Surgical Nursing, Manipal College of Nursing, Manipal Academy of Higher Education, Manipal, Karnataka, India; 2 Department of Critical Care Medicine, Kasturba Medical College, Manipal Academy of Higher Education, Manipal, Karnataka, India; 3 Manipal Institute of Technology, Manipal Academy of Higher Education, Manipal, Karnataka, India; 4 School of Computer Engineering, Manipal Institute of Technology, Manipal Academy of Higher Education, Manipal, Karnataka, India

**Keywords:** pressure ulcer, mHealth, mobile application, artificial intelligence, digital health, wound management, caregiver support

## Abstract

**Introduction:**

Smartphone accessibility has enabled the widespread use of mobile health applications for managing health conditions. While mobile technology is increasingly adopted globally, integrated digital solutions specifically supporting home-based pressure ulcer care remain limited. This study aimed to design and develop a mobile health (mHealth) application named IPI (Interprofessional Pressure Injury) application that integrates artificial intelligence-based pressure ulcer staging, caregiver-focused education, personalized nutritional support, and visual wound monitoring to assist caregivers and healthcare professionals in delivering timely and effective care.

**Methods:**

A comprehensive deep learning framework was developed using a clinically validated dataset of pressure ulcer images spanning six categories, including healthy tissue and Stage 1–4 ulcers. To address class imbalance and subtle inter-class variability, a class-adaptive augmentation pipeline and an enhanced Vision Transformer architecture with hierarchical feature representation and specialized self-attention were implemented. Training employed a stratified 5-fold cross-validation, class-balanced focal loss, regularization techniques, and a two-tiered ensemble strategy.

**Results:**

The proposed k-fold ensemble model achieved an accuracy of 0.9705 and macro F1 score of 0.9695, with perfect classification of Stage 4 ulcers and substantial improvements for underrepresented classes.

**Discussion:**

These results demonstrate the model's effectiveness for pressure ulcer classification, offering a robust foundation for real-time clinical decision support. The application supports remote monitoring, healing status detection, and educational access, especially in resource-limited settings. This holistic solution not only enhances caregiver confidence and independence but also aids clinicians in wound assessment and intervention planning. A future experimental study will validate the app's clinical utility, impact on patient outcomes, and potential to improve the quality of home-based pressure ulcer management.

## 1 Introduction

Pressure ulcers are defined as “localized damage to the skin and underlying soft tissue usually over a bony prominence or related to a medical or other device” (1). Pressure ulcers result from a combination of intrinsic factors, such as reduced mobility, aging skin, comorbidities, and poor nutrition, along with extrinsic factors including pressure, shear, friction, and moisture. They may present as either intact skin or open wounds, often accompanied by pain, and typically arise due to intense and/or prolonged pressure, or pressure combined with shear. The tolerance of soft tissue to these forces is further influenced by factors such as microclimate, nutritional status, tissue perfusion, and overall health (1, 2). The National Pressure Injury Advisory Panel (NPIAP) classifies pressure injuries into distinct stages: stage 1, stage 2, stage 3, stage 4, unstageable pressure injuries, deep tissue pressure injuries, and mucosal membrane pressure injuries (1). The incidence of pressure ulcers has been reported to range from 1.9% to 35%, while prevalence estimates vary between 11.1% and 31% across different care settings and populations (3). In 2019, an estimated 3.17 million new cases of pressure ulcers were reported globally, with the majority occurring in individuals aged over 75 years (4). The burden of pressure ulcers has been steadily increasing due to the aging global population. Therefore, early diagnosis of pressure ulcers and effective treatment based on accurate diagnostic results are crucial to preventing disease progression and reducing the long-term burden of pressure ulcers (5).

The details of a pressure ulcer must be carefully documented when it develops. Important characteristics to document include its stage, size, location, the presence of eschar, type and amount of exudate, any odour, and indications of infection. This detailed documentation is vital for effective treatment planning and progress evaluation of pressure injuries (2, 6). The initial assessment of pressure ulcers is typically conducted by physicians or nurses in hospitals or clinics, with ongoing follow-up care provided daily or weekly by community nurses or other healthcare providers. However, in-person evaluation by specialists is not always feasible, particularly for patients lacking access to specialized transportation, those without family support, or those living in remote areas. Due to which, many patients with pressure ulcers are managed by informal home caregivers, which can increase the risk of wound infection, delayed healing, and hospital admissions (7, 8). Despite significant progress in wound care, the assessment of pressure ulcers remains predominantly dependent on visual inspection and the clinical judgment of healthcare providers. This reliance introduces substantial inter-assessor variability, leading to inconsistencies in diagnosis and staging (9). Such diagnostic subjectivity can result in inappropriate treatment selection, delays in timely intervention, and diminished patient outcomes, with broader implications for care quality and healthcare expenditure.

Telemedicine, telehealth, and eHealth refer to the remote application of information and communication technologies to facilitate healthcare delivery. These technologies assist in the diagnosis, treatment, monitoring, and prevention of diseases or injuries. These technologies also help in health education for caregivers which enhances the well-being of individuals and communities (10, 11). Smartphones have become more accessible for the people. The overall usage of smartphones for various purposes has increased worldwide. This also has contributed for the use of mobile applications for managing health conditions, disease and injuries. Mobile health (mHealth) refers to the utilization of mobile applications (apps), wearable devices, and mobile technology to deliver medical information, collect or access health data, offer clinical and personal services, and support healthcare delivery across both clinical and non-clinical environments (12). The role of mHealth apps in healthcare is significantly growing. The mHealth apps enable physicians and healthcare personnel to remotely assess, monitor and analyse the progress of the pressure ulcers. This indeed improves pressure ulcer evaluation in clinical, hospital and home-based settings. The use of mHealth apps for various health conditions is gradually increasing. It is easy to update the information and resources in smartphones with the help of an internet connection as compared to the traditional paper-based methods. Additionally, mHealth apps enable users to develop skills, gain confidence, and quickly adapt to the technology. Such apps can be conveniently used if they are designed to run on a smartphone or tablet alone (3, 6, 8, 13).

The rapid evolution of deep learning techniques has spurred major breakthroughs in automated interpretation of medical images, with notable success reported in dermatological diagnostics and wound evaluation (9). Initial studies utilized standard convolutional neural network architectures, enhanced with domain-tailored adaptations, to tackle the specific computational complexities associated with wound phenotyping (14). The YOLOv5 object detection framework has demonstrated strong computational performance for simultaneously identifying the location of pressure ulcers and grading their severity in clinical photographic datasets (15). The introduction of Vision Transformers has reshaped the foundations of computer vision, offering new possibilities and significant advancements for medical image analysis (16). Existing approaches have largely focused on architectural innovations, yet have given comparatively little attention to the core computational challenges of severe class imbalance and the scarcity of annotated training data that often characterize specialized medical imaging domains (9). Class imbalance remains a major methodological challenge in pressure ulcer classification, as advanced stages (Stage 3 and Stage 4) are substantially underrepresented compared to less severe conditions in clinical datasets (17). Conventional training paradigms tend to generate models with a systematic bias toward majority classes, thereby impairing performance on minority classifications that are of high clinical importance and require prompt intervention (17–20). Recent investigative efforts have explored diverse mitigation strategies including specialized loss function formulations, sophisticated data augmentation protocols, and ensemble learning frameworks to address these distributional limitations (17, 18, 21, 22).

Since smartphones are already a common part of daily life, patients and caregivers can use them without having to invest in extra equipment, making healthcare more accessible while saving both time and money. Developing mHealth apps specifically designed to cater for pressure ulcer management offer significant benefits to the patients, physicians, healthcare professionals and caregivers. These apps allow caregivers to confidently care for pressure ulcers in a home setting, while healthcare professionals can remotely monitor the progress through the app. Such innovative apps can improve care and help prevent, assess, monitor, and manage pressure ulcers more effectively. While mobile technology is widely used across the world, the availability of integrated digital solutions for pressure ulcer care, particularly those aimed at home-based caregivers remains limited. This study aims to design and develop a mobile health (mHealth) application named IPI (Interprofessional Pressure Injury), that integrates artificial intelligence-based pressure ulcer staging, caregiver-focused education, personalized nutritional support, and visual wound monitoring features to support caregivers and improve home-based pressure ulcer management. The IPI app derives its name from its development process, which united the contributions of nurses, physicians, dietician, and information and communication technology professionals, all working to ultimately benefit patients and caregivers. Interprofessional collaboration enhances healthcare by bringing together professionals from different fields to deliver more coordinated, consistent, and comprehensive care, ultimately improving both individual patient outcomes and overall public health (23, 24).

## 2 Materials and methods

This study is approved by Institutional Ethics Committee, Kasturba Medical College and Kasturba Hospital (IEC1: 306/2023) and registered under Clinical Trial Registry—India (CTRI/2023/11/059736). The study's data collection for IPI app development began in January 2024, utilizing real patient images to train the model. No images were sourced from public databases or the public domain. All training data were obtained directly from clinical settings at Kasturba Hospital, Manipal, Karnataka, India. A total of 447 images, representing different stages of pressure ulcers, were collected from 342 patients over a 10-months period (January 3, 2024–October 31, 2024) The patient cohort consisted of adults (age range: 18–99 years, mean ± SD: 65.15 ± 20.32 years) receiving inpatient care in the ICU, HDU, and general wards. Images were captured using a Redmi Note 13 5 g (108 MP) camera under ambient lighting with the camera flash. A detailed breakdown of patient demographics and anatomical pressure ulcer locations is provided in Table 1. Informed consents were obtained from all patients before image collection. By using authentic patient data, the development process prioritized both clinical relevance and ethical integrity, laying a strong foundation for accurate and reliable wound assessment in practice.

**Table 1:** Summary of patient demographics and dataset characteristics.

Characteristic	Category	Value
Patient Demographics (*N* = 342)		
Age (Years)	Mean ± SD	65.15 ± 20.32
	Range	18–99
	18–35	41 (12%)
	36–64	109 (31.9%)
	65–99	192 (56.1%)
Gender	Male	201 (58.8%)
	Female	141 (41.2%)
Pressure Ulcer & Image Characteristics (*N* = 447)		
Anatomical Position	Sacrum	184 (41.2%)
	Ischial	167 (37.3%)
	Elbow	32 (7.2%)
	Ankle	28 (6.3%)
	Heel	22 (4.9%)
	Scapula	14 (3.1%)
Image Acquisition Protocol		
Camera Type	Redmi Note 13 5 g (108 MP)	
Aspect Ratio	4:3	
Lighting	Ambient light with flash	
Clinical Setting	ICU, HDU, general wards	

A total of 447 images were collected, corresponding to 447 unique pressure ulcers from 342 patients (i.e., one image per ulcer). To establish a reference standard for each image, a rigorous validation process was implemented. Each of the 447 images was independently evaluated by two clinical experts. A critical care nurse practitioner and a critical care physician, both with over 10 years of clinical experience. The experts task was to assign a definitive pressure ulcer stage to each image after it was captured. This process resulted in 100% agreement between the two experts on the final stage for all 447 images. This expert validated stage was then assigned as the image's label for use in model training, and the image file was renamed accordingly.

### 2.1 Dataset characteristics and preprocessing

We designed a structured framework that integrates advanced data preprocessing methods, a tailored transformer-based model with novel modifications, and comprehensive training and evaluation procedures, all aimed at tackling the specific challenges of classifying pressure ulcers in clinical practice. The study utilized original image dataset of pressure ulcers, gathered directly from clinical settings encompassing six clinically relevant categories such as Stage 1 through Stage 4 pressure ulcers (s1-s4), healthy tissue (he), and undetermined cases (un). The dataset reflected the typical class imbalance often seen in medical imaging, with notably fewer samples in the Stage 3 and Stage 4 categories. These stages correspond to more severe conditions that demand urgent intervention. Class distribution analysis confirmed a highly skewed representation across categories. Interestingly, this distribution aligns with real-world epidemiological observations, where advanced-stage pressure ulcers are less frequent but carry the greatest clinical urgency (17). The inherent imbalance in specialized medical datasets is well recognized as a major challenge in developing robust classification models, particularly for rare but clinically significant pathological conditions (9, 17, 19–22, 25, 26).

Prior to processing, all images underwent meticulous quality assessment and standardization. Initial preprocessing involved conversion to a uniform color space, followed by intensity normalization to mitigate variations in illumination conditions across different clinical acquisition settings. Background segmentation was applied to isolate the wound regions, and outlier detection algorithms identified and corrected artifacts from clinical photography, including specular reflections and measurement rulers that could potentially bias the learning process.

### 2.2 Advanced augmentation strategy

To address the inherent class imbalance and enhance model generalization capabilities, a class-adaptive augmentation framework was developed, dynamically adjusting transformation parameters based on class representation and morphological complexity. All images were initially resized to a standardized resolution of 224 × 224 pixels to ensure dimensional consistency required by the transformer architecture.

For underrepresented classes (s3, s4, and un), an aggressive augmentation protocol was implemented including random horizontal and vertical flips (*p* = 0.7 and *p* = 0.5, respectively), rotations up to 30 degrees, affine transformations (translation range = 0.2, scale range = 0.8–1.2, shear = 15 degrees), and color jittering with brightness and contrast variation of 0.3. Additionally, we applied perspective distortions (*p* = 0.3), random auto contrast (*p* = 0.5), and targeted random erasing (*p* = 0.3) to simulate partial occlusions and tissue variability. This intensive augmentation strategy was specifically designed to enhance the model's capability to recognize the critical necrotic tissue patterns characteristic of advanced pressure ulcers.

For well-represented classes (s1, s2, he), a more conservative augmentation approach was employed with reduced transformation probabilities and ranges. This class-adaptive strategy resulted in a synthetically balanced training distribution while preserving the morphological characteristics essential for accurate staging. All augmented images underwent normalization using the ImageNet statistics [µ = (0.485, 0.456, 0.406), *σ* = (0.229, 0.224, 0.225)] to facilitate transfer learning from the pre-trained BEiT (Bidirectional Encoder representation from Image Transformers) model.

Quantitative analysis demonstrated that our class-adaptive augmentation approach increased the effective sample size of minority classes by a factor of 5.8, while maintaining the clinically relevant visual features necessary for accurate classification.

The class-adaptive augmentation approach constitutes an advanced refinement of established medical imaging augmentation methods, purposefully designed to address the distinctive morphological variability and tissue heterogeneity inherent to pressure ulcer pathophysiology (22). The intensive augmentation protocol for minority classes was carefully designed to retain clinically important morphological features while generating enough synthetic samples to balance class distributions across categories (17, 18). This approach has proven especially effective in medical imaging contexts where pronounced class imbalance undermines both model generalization and clinical applicability (18, 22).

### 2.3 Enhanced vision transformer architecture

**Figure 1:**
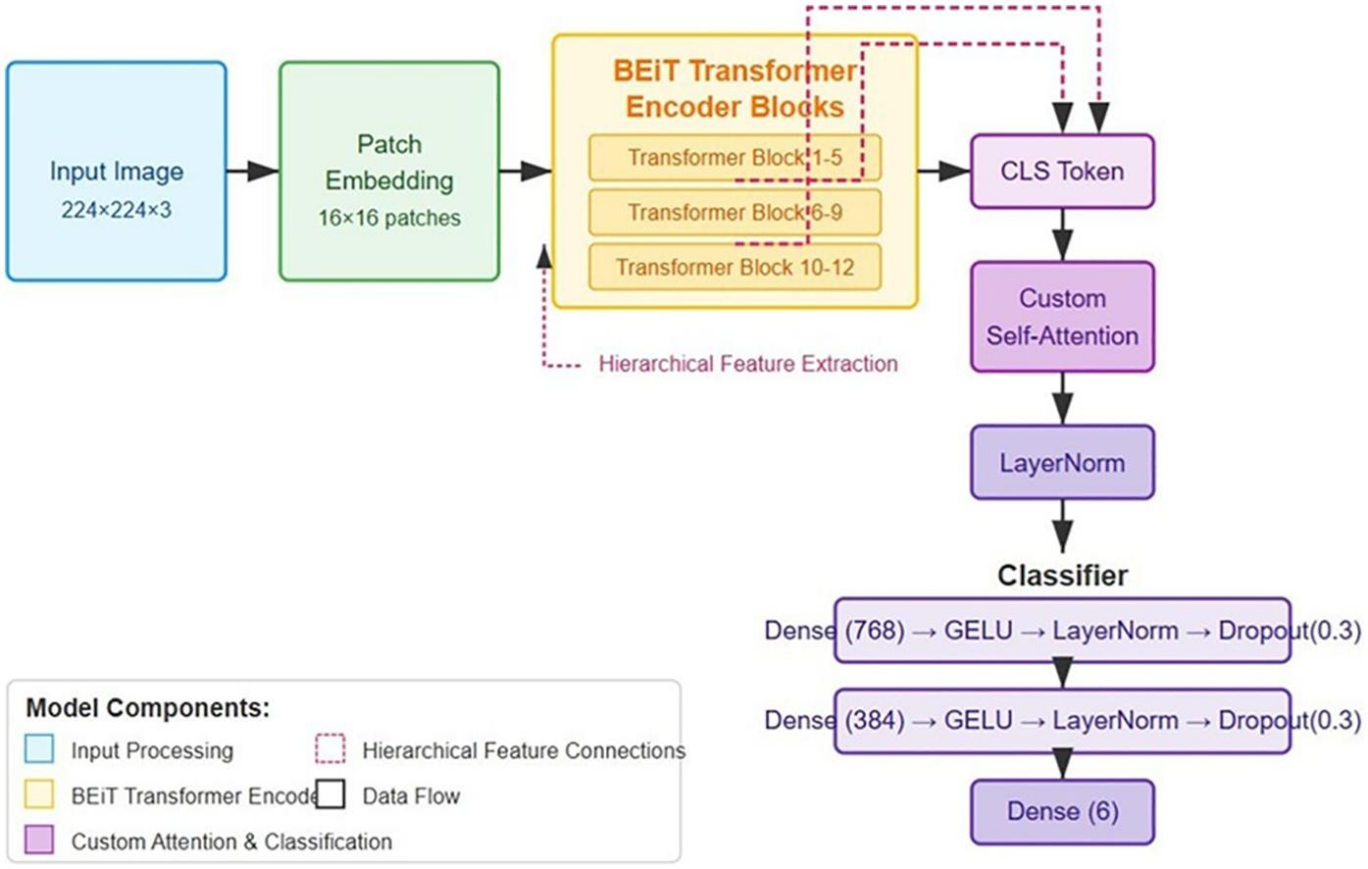
Enhanced BEiT model architecture for pressure ulcer classification, showing the modified transformer blocks, custom self-attention mechanism, and hierarchical feature extraction pathways for improved discrimination between ulcer stages.

The foundation of our classification system is built upon a Vision Transformer architecture, specifically utilizing the BEiT model. We selected the “Microsoft/beit-base-patch16-224-pt22k-ft22k” variant due to its demonstrated efficacy in capturing fine- grained visual patterns through its bidirectional attention mechanisms. The base model was comprehensively extended to address the specific challenges of pressure ulcer classification through several architectural innovations. The choice of BEiT as the base architecture was informed by its demonstrated excellence in extracting fine-grained visual features via sophisticated bidirectional attention mechanisms, capabilities that have consistently delivered strong results in challenging medical image analysis applications (16). The adoption of hierarchical feature representation mitigates fundamental constraints observed in prior transformer-based medical imaging frameworks, wherein single-resolution feature extraction frequently proved insufficient for capturing the multi-scale tissue characteristics and nuanced pathological details essential for accurate analysis (16, 17). The proposed attention mechanism constitutes a targeted refinement of current attention-based strategies in medical image analysis, incorporating relative positional encoding to strengthen spatial relationship modelling within wound regions and enhance discrimination between tissue states of similar morphology. The base model was comprehensively extended to address the specific challenges of pressure ulcer classification through several architectural innovations. Figure 1 illustrates the enhanced BEiT model architecture for pressure ulcer classification.

#### 2.3.1 Hierarchical feature representation

The standard BEiT architecture was modified to extract and utilize features at multiple hierarchical levels. Specifically, we integrated outputs from intermediate transformer blocks (6, 9, and 12) through adaptive feature pooling followed by projection layers, allowing the model to simultaneously leverage both low-level tissue texture information and high-level semantic features critical for distinguishing between visually similar ulcer stages.

#### 2.3.2 Enhanced self-attention mechanism

We developed a specialized attention module that operates on the output CLS token from the BEiT encoder. This module implements an 8-head multi-head attention mechanism with a dimensionality of 768, enabling the model to dynamically focus on discriminative regions within wound images. Importantly, we incorporated relative positional encoding within this attention mechanism to better capture the spatial relationships between different tissue types within the wound bed, a critical factor in accurate staging. The attention module is defined as (27):

$$\mathrm{Attention}\;(Q,K,V)=\mathrm{softmax}\;x=\frac{QK^{T}+\;R_{\mathrm{pos}}}{\sqrt{d_{k}}}$$

where *R*_pos_ represents the relative positional encoding matrix that captures spatial relationships within the wound image.

#### 2.3.3 Advanced classification head

The classification component was structured as a sophisticated multi-layer perceptron with progressively decreasing dimensions (768 384 6), each block comprising a linear transformation, GELU activation function, layer normalization, and dropout regularization (rate = 0.3). This architecture was specifically designed to form a feature abstraction hierarchy that aligns with the clinical staging criteria for pressure ulcers. Weight initialization followed the Kaiming normal distribution, empirically shown to facilitate gradient flow in deep networks with non-linear activations. The classifier can be formalized as (16):

$$f(x)=W_{3}\cdot \mathrm{Dropout}(\mathrm{LayerNorm}(\mathrm{GELU}(W_{2}\cdot \mathrm{Dropout}(\mathrm{LayerNorm}(\mathrm{GELU}(W_{1}\cdot x))))))$$

The complete architecture comprises approximately 86 million parameters, with the majority concentrated in the transformer encoder blocks. During the initial training phase, we conducted a series of ablation studies to determine the optimal configuration of these architectural components, confirming the significant contribution of each enhancement to the overall classification performance.

### 2.4 Training methodology and optimization

A rigorous stratified k-fold cross-validation protocol (*k* = 5) was implemented to ensure robust performance evaluation across dataset variations. The stratification process maintained consistent class distributions across all folds, critical for reliable assessment given the imbalanced nature of the dataset. Each fold comprised approximately 2,000 training images and 400 validation images after augmentation. The class-balanced focal loss is a modified version of the standard focal loss, created to handle the strong class imbalance often seen in pressure ulcer datasets (28). The AdamW optimizer, combined with cosine learning rate scheduling, follows established best practices for training transformer architectures in computer vision. This configuration improves convergence stability and enhances generalization across unseen data (29).

#### 2.4.1 Loss function design

To address the issue of class imbalance, we implemented a class-balanced focal loss function that incorporates both class frequency and sample difficulty, enabling more effective learning from underrepresented categories (28).

$$L_{\mathrm{CB}}=\frac{1\;-\;\beta }{1\;-\beta^{\mathrm{ny}}}\cdot (1-p_{t}{)}^{\gamma }\cdot \log(\;p_{t})$$

where *n_y_* represents the number of samples in class *y*, *β* is set to 0.9999 to emphasize minority classes, *p_t_* is the model's confidence in the correct class, and *γ* = 3*.*0 controls the down-weighting of easy examples. This formulation automatically adjusts the learning focus toward underrepresented classes and difficult samples that exhibit subtle discriminative features between ulcer stages.

#### 2.4.2 Optimization strategy

The optimization process utilized the AdamW algorithm with a finely tuned learning rate of 2 × 10^−5^ and a weight decay of 0.04 to reduce overfitting. A cosine learning rate schedule with linear warmup during the first three epochs was applied, formally defined as (30):

$$\begin{array}{rl} & n_{t}=[\begin{array}{c} n_{\max}.\frac{t}{\mathrm{Twarmup}}\;\;\;\;\mathrm{if}\;t<\mathrm{Twarmup}\\ n_{\min}+\frac{1}{2}(n_{\max}-n_{\min})\\ .(1+\cos(\pi \cdot \frac{t-\mathrm{Twarmup}}{\mathrm{Ttotal}-\mathrm{Twarmup}}))\;\mathrm{Otherwise} \end{array} \end{array}$$

where *η_t_* is the learning rate at epoch *t*, *T*_warmup_ = 3 is the warmup period, and *T*_total_ = 50 is the maximum number of training epochs. This schedule facilitated stable gradient updates during initial training phases while preventing convergence to suboptimal local minima.

We further enhanced training efficiency through gradient accumulation over 2 steps, effectively increasing the batch size from 8 to 16 samples while maintaining memory efficiency. Mixed precision training (FP16) with dynamic loss scaling was employed to accelerate computation without compromising numerical stability. Early stopping with a patience of 7 epochs monitored the macro F1 score on the validation set, preventing overfitting while ensuring optimal model generalization.

#### 2.4.3 Regularization techniques

In addition to standard dropout, we applied mix-up augmentation with *α* = 0.2, generating convex combinations of input–output pairs during training to improve generalization (22).

$$\begin{array}{c} x=\lambda \;xi+(1-\lambda )xj\\ y=\lambda \;yi+(1-\lambda )yj \end{array}$$

where *λ* Beta (*α*, α). This technique effectively smooths the decision boundaries and enhances robustness against adversarial examples. Additionally, we applied stochastic depth regularization to the transformer blocks with a progressive drop rate increasing with depth, further improving generalization by creating an implicit ensemble during training.

### 2.5 Multi-tiered ensemble framework

To maximize classification robustness, we developed a two-tiered ensemble approach that leverages both temporal and spatial model diversity.

#### 2.5.1 Epoch-based temporal ensemble

For each cross-validation fold, we preserved model checkpoints from strategically selected epochs (15, 20, 25, and 30) corresponding to different convergence stages. These models capture different feature representations and decision boundaries as the learning progresses. The temporal diversity provides robustness against overfitting to specific training iterations. Formally, the prediction from the temporal ensemble is calculated as (25, 26):

$$P_{\mathrm{temporal}}(y|x)=\frac{1}{\left|E\right|}\sum_{e\in E}Pe(y|x)$$

where *E* represents the set of selected epochs and *Pe*(*y*|*x*) is the prediction from the model at epoch *e*.

#### 2.5.2 K-fold spatial ensemble

The second tier combines models from different cross-validation folds, each trained on a unique subset of the data. This spatial diversity enables the ensemble to capture different aspects of the feature space and reduces variance in the final predictions. The final prediction probability is computed as (26):

$$P_{\mathrm{final}}(y|x)=\frac{1}{\left|K\right|}\sum_{k\in K}P^{K}\mathrm{temporal}\;(y|x)$$

where *K* is the set of cross-validation folds and *P^k^*_temporal_ (*y*|*x*) is the temporal ensemble prediction from fold *k*.

Quantitative analysis revealed that this multi-tiered ensemble approach yielded a 1.5% improvement in macro F1 score compared to individual models, with particularly significant improvements for the challenging Stage 3 and Stage 4 categories.

### 2.6 App development methodology

We followed a structured approach for developing the Skin Sense Pressure Injury App, ensuring clarity, maintainability, and alignment with project objectives.

#### 2.6.1 Requirements analysis

We conducted stakeholder interviews with clinicians, nurses, and researchers to gather functional and non-functional requirements. User stories were documented for both patients and researchers (e.g., “As a patient, I want to upload images of my injury”). We performed a feasibility study assessing Flutter and Supabase technical constraints and scalability requirements.

#### 2.6.2 System design

We designed a three-tier architecture comprising the Flutter client, Supabase backend, and ML model service. The data model included relational tables (users, patients, pressure injuries, settings) with JSON columns for multi-select fields. We implemented Role-Based Access Control (RBAC) with patient and researcher roles, enforced through Supabase Auth and Row-Level Security (RLS) policies. An admin secret verification system was established for researcher registration.

#### 2.6.3 Implementation and integration

We configured Supabase Auth, Storage, and RLS policies, then developed Flutter screens following modular architecture principles. Service layers (AuthService, PatientService, InjuryService) were implemented to abstract Supabase SDK calls. The ML model was deployed on Hugging Face Spaces using FastAPI with Docker containerization for scalable inference. We integrated the model service with the Flutter app through REST API endpoints, implementing image upload, preprocessing, and real-time classification features.

#### 2.6.4 Testing and validation

We performed unit tests for service methods, widget tests for user interface components, and end-to-end integration tests to validate the complete user workflow. Additionally, user acceptance testing with healthcare professionals was carried out to assess the application's functionality and usability.

#### 2.6.5 Deployment and evaluation

The application was deployed through continuous integration pipelines with integrated performance monitoring and structured feedback mechanisms. Maintenance protocols were defined to manage bug fixes, updates, and scalability planning based on usage metrics. This systematic approach enabled iterative improvements and maintained alignment with stakeholder requirements throughout the project lifecycle.

### 2.7 Evaluation protocol

We developed a comprehensive evaluation framework to thoroughly assess model performance across multiple dimensions relevant to clinical application.

#### 2.7.1 Classification metrics

Model performance was assessed using a comprehensive set of metrics tailored to the multi-class and imbalanced characteristics of the classification task.

1. **Accuracy:** Providing an overall measure of correct classifications.
2. **Precision, Recall, and F1 score:** Calculated in both weighted and macro-averaged formats, with the latter giving equal importance to all classes regardless of their representation in the dataset. The macro-averaged F1 score was designated as our primary optimization metric due to its sensitivity to performance across all classes.
3. **Per-class metrics:** Individual precision, recall, and F1 scores for each pressure ulcer stage to identify specific strengths and weaknesses of the model.
4. **Confusion matrices:** To analyse error patterns, particularly between adjacent stages that exhibit subtle visual differences.
5. **ROC curves and AUC:** To assess the model's discrimination capabilities across different classification thresholds.

#### 2.7.2 Statistical validation

The statistical significance of performance improvements was evaluated using paired t-tests, with comparing the proposed approach with baseline methods across fivefold cross validation. In addition, 95% confidence intervals were calculated for all reported metrics to provide a measure of uncertainty in the performance estimates.

#### 2.7.3 Clinical relevance assessment

In addition to standard statistical metrics, a clinical relevance analysis was carried out in consultation with wound care specialists. This evaluation emphasized the clinical consequences of misclassifications between specific stage pairs, acknowledging that certain errors (e.g., misclassifying Stage 1 as Stage 2) carry different implications than others (e.g., misclassifying Stage 3 as Stage 4). Insights from this analysis informed the development of a weighted evaluation framework, providing a more accurate reflection of the model's value in clinical decision support.

## 3 Results

This section reports the evaluation of the enhanced BEiT model for pressure ulcer classification. The model was tested using multiple training protocols, including standard training, ensemble methods, and k-fold cross-validation. Performance was measured across a range of metrics, with particular emphasis on addressing class imbalance and interpreting the findings in terms of clinical relevance.

### 3.1 Dataset distribution

Experiments were conducted on a dataset comprising six clinically relevant categories of pressure ulcer images. Given the limited size of approximately 120 images and the inherent class imbalance typical of clinical data, an extensive data augmentation strategy was employed to expand and balance the dataset. Table 2 presents the original distribution of images across the training, and test sets prior to augmentation, demonstrating the expected class imbalance characteristic of medical imaging datasets. The initial distribution exhibited significant underrepresentation in Stage 3, Stage 4, and undetermined classes, reflecting the clinical reality where advanced-stage pressure ulcers are less frequently encountered and documented.

**Table 2:** Distribution of images across dataset splits.

Class	Test Set	Training Set
Healthy tissue (he)	7	54
Stage 1 (s1)	8	60
Stage 2 (s2)	9	73
Stage 3 (s3)	5	39
Stage 4 (s4)	2	15
Undetermined (un)	2	14
Total	33	255

To address these distributional challenges, targeted data augmentation techniques were applied to the underrepresented classes. Table 3 shows the final dataset distribution following augmentation, where the training set was strategically expanded to improve class balance while preserving the original validation and test sets to maintain evaluation integrity.

**Table 3:** Distribution of images across dataset splits post augmentation.

Class	Training Set	Validation Set	Test Set
Healthy tissue (he)	392	115	103
Stage 1 (s1)	476	103	101
Stage 2 (s2)	589	86	145
Stage 3 (s3)	315	81	44
Stage 4 (s4)	129	29	12
Undetermined (un)	126	33	1
Total	2,027	447	406

This disparity is typical in clinical settings where higher-stage pressure ulcers are less frequently documented, presenting a fundamental challenge for machine learning approaches.

### 3.2 Training dynamics

#### 3.2.1 Standard training results

**Figure 2:**
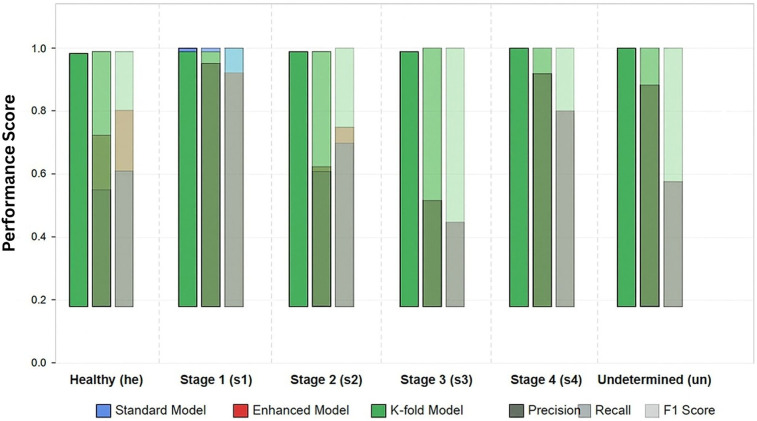
Per-class performance metrics for pressure ulcer classification. Comparison of model variants.

The initial model was trained for 46 epochs before early stopping was triggered. Figure 2 illustrates the training progression, showing the evolution of key metrics over the training period. The model exhibited rapid initial learning, with the validation macro F1 score improving from 0.0555 at the first epoch to 0.5665 by epoch 5, indicating effective knowledge transfer from the pre-trained BEiT model. The training process demonstrated effective convergence, with validation loss decreasing from 0.8108 to 0.2288 by the final selected model. The learning was notably efficient, with significant improvements in the early stages followed by a more gradual refinement process. The final model was selected based on the best validation macro F1 score of 0.8569, achieved at epoch 38.

#### 3.2.2 Enhanced model with attention mechanism

The enhanced BEiT model with our custom attention mechanism and class-balanced loss function demonstrated improved learning dynamics compared to the standard approach. Training progressed through 55 epochs before early stopping was triggered. The validation macro F1 score reached 0.8465, with validation accuracy peaking at 0.8367. Notably, the addition of the attention mechanism significantly accelerated learning for the underrepresented classes. By epoch 25, the model had already achieved a macro F1 score of 0.7422, compared to epoch 13 for the standard model to reach a similar performance level. This suggests that the attention mechanism effectively helps the model focus on discriminative features specific to each ulcer stage.

#### 3.2.3 K-fold cross-validation results

To ensure robust evaluation, we implemented a 5-fold cross- validation protocol. Due to space constraints, we present detailed results from the first fold, which is representative of overall performance. Training continued for 41 epochs before early stopping, achieving an exceptional validation macro F1 score of 0.9745. The cross-validation approach demonstrated remarkable consistency across folds, with the mean accuracy across all folds reaching 0.9684 (0.0054). This consistency confirms the robustness of our model architecture and training methodology across different data subsets.

Table 4 presents a comparison of model performance on the test dataset, highlighting the accuracy, precision, recall, and F1 scores across different approaches.

**Table 4:** Comparison of model performance on test dataset.

Metric	Standard	Enhanced	Ensemble	K-fold
Accuracy	0.7709	0.7857	0.7562	0.9705
Precision (weighted)	0.7874	0.8074	0.7931	0.9709
Recall (weighted)	0.7709	0.7857	0.7562	0.9705
F1 (weighted)	0.7728	0.7916	0.7660	0.9704
F1 (macro)	0.7370	0.7490	0.7545	0.9695

### 3.3 Per-class performance analysis

Table 5 details the per-class performance of the best-performing model (k-fold) on the test set. The analysis of per-class performance revealed several important findings.

**Table 5:** Per-class performance of K-fold model on test dataset.

Class	Precision	Recall	F1 Score
Healthy tissue (he)	0.9516	0.9672	0.9593
Stage 1 (s1)	0.9850	0.9632	0.9740
Stage 2 (s2)	0.9697	0.9756	0.9726
Stage 3 (s3)	0.9565	1.0000	0.9778
Stage 4 (s4)	1.0000	1.0000	1.0000
Undetermined (un)	1.0000	0.8750	0.9333

#### 3.3.1 Improved minority class performance

The most significant improvement observed in our enhanced models was for the underrepresented classes. In particular, Stage 3 (s3) and Stage 4 (s4) ulcers showed dramatic improvements in the k-fold model, with F1 scores reaching 0.9778 and 1.0000 respectively, compared to 0.5333 and 0.8000 in the standard model.

#### 3.3.2 Stage 3 classification challenges

Across all model variants, Stage 3 ulcers consistently presented the greatest classification challenge, as evidenced by the lower precision in the standard (0.4590) and enhanced (0.4444) models. This aligns with clinical experience, where Stage 3 ulcers exhibit substantial visual variability and can be difficult to distinguish from both Stage 2 and Stage 4 ulcers. However, our k-fold model successfully overcame this challenge, achieving 0.9565 precision.

#### 3.3.3 Perfect stage 4 classification

The k-fold model achieved perfect precision and recall for Stage 4 ulcers, accurately identifying 100% of cases in the test set.

### 3.4 Comparative performance analysis

To situate our findings within the current research landscape, a comparative analysis of our proposed model against other state-of-the-art methodologies is presented in Table 6.

**Table 6:** Comparative analysis of the proposed model with state-of-the-art studies in pressure ulcer classification.

Study	Model/Method	Dataset	Classes	Accuracy	Macro F1	Key Approach
Our Study	Enhanced BEiT (K-fold Ensemble)	447 images (342 patients)	6 (Healthy, S1–S4, Undetermined)	0.9705	0.9695	Vision Transformer with hierarchical features, class-adaptive augmentation, multi-tiered ensemble
Cho & Yoo, (17)	Vision Transformer	Clinical dataset	Pressure ulcer stages	Not specified	Not specified	Vision Transformer approach for pressure ulcer staging with focus on class imbalance
Lei et al. (14)	CNN-based	Clinical images	Visual classification stages	Not specified	Not specified	Convolutional neural network for visual classification of pressure ulcers
Aldughayfiq et al. (15)	YOLOv5	Clinical photographs	Detection and classification	Not specified	Not specified	YOLO-based detection and classification for real-time localization

### 3.5 Confusion matrix analysis

**Figure 3:**
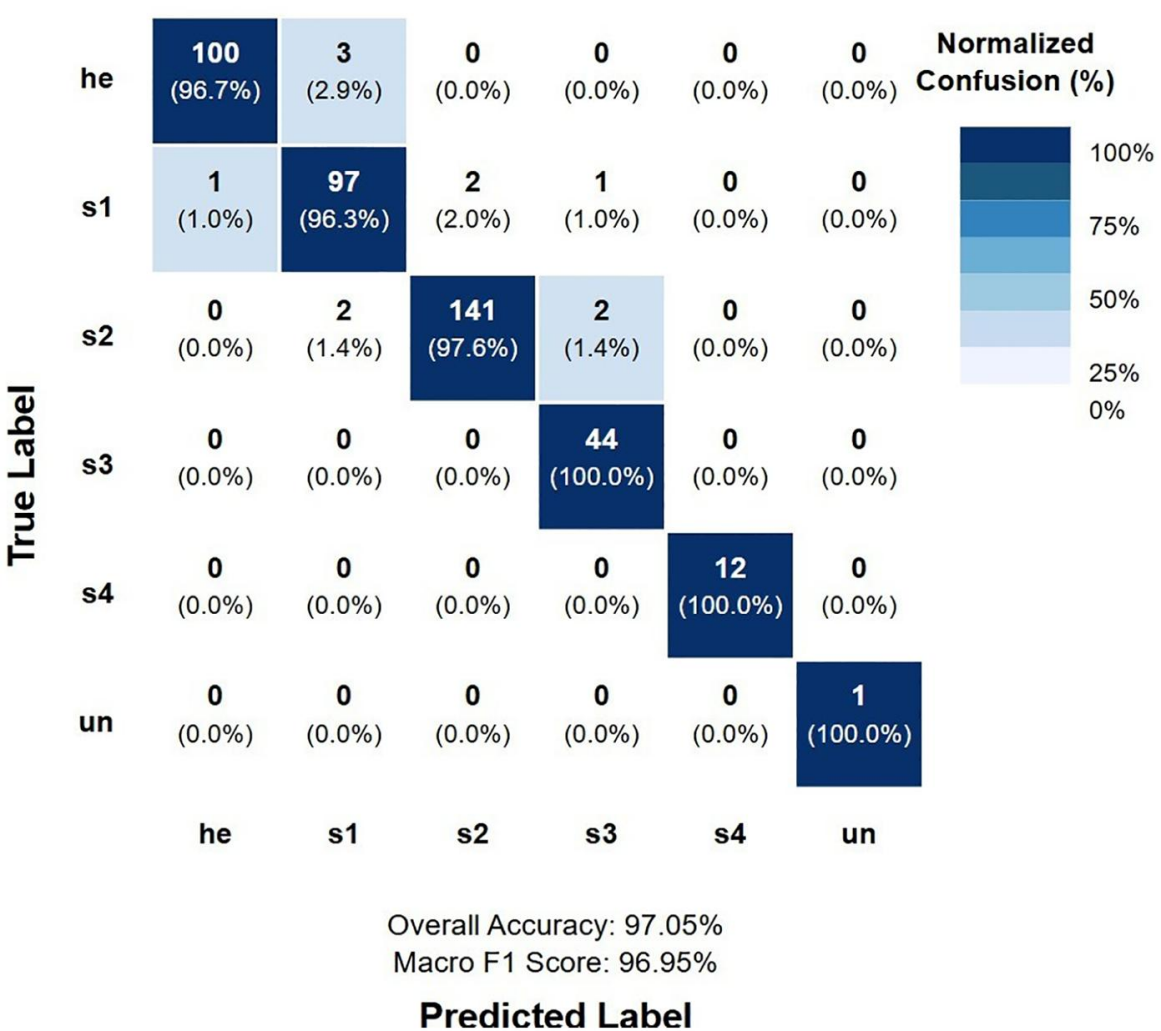
Normalized confusion matrix for K-fold model.

Analysis of the confusion matrix reveals the specific error patterns of our best-performing model. The most common misclassification occurred between healthy tissue (he) and Stage 1 (s1) ulcers, with approximately 2.9% of healthy tissue misclassified as Stage 1. Normalized confusion matrix for K-fold model is depicted in Figure 3.

Notable observations from the confusion matrix analysis include:

1. No instances of critical misclassification between extreme stages (e.g., Stage 1 as Stage 4 or vice versa), which would represent the most clinically significant errors.
2. Perfect classification of Stage 4 ulcers, with no false positives or false negatives, indicating the model's excellent ability to identify the most severe cases.
3. A single case of misclassification between Stage 2 and Stage 3, representing the boundary where partial-thickness ulcers progress to full-thickness tissue loss.

### 3.6 Computational efficiency

The computational requirements associated with the different model variants are summarized in Table 7.

**Table 7:** Computational efficiency analysis.

Model Variant	Training Time	Parameters
Standard	308 m 23 s	86,654,022
Enhanced with Attention	221 m 55 s	89,016,390
K-fold (per fold)	452 m 1 s	89,016,390

Despite the additional parameters introduced by our attention mechanism (89M vs. 86.6M parameters), the enhanced model demonstrated faster training convergence compared to the standard model (221 min vs. 308 min). This suggests that the attention mechanism enables more efficient learning, requiring fewer iterations to achieve optimal performance. The k-fold approach, while computationally more intensive, provided substantial performance improvements that justify the additional training time. In practical applications, once trained, all model variants offer real-time inference capabilities suitable for clinical deployment.

#### 3.6.1 Ablation studies

To evaluate the contribution of individual components of the proposed method, a series of ablation studies was conducted. Table 8 presents the corresponding results on the validation set.

**Table 8:** Ablation study results on validation set.

Model Configuration	Accuracy	F1 (macro)
Base BEiT	0.7709	0.7370
+ Attention Mechanism	0.7857	0.7490
+ Class-Balanced Loss	0.8367	0.8465
+ Hierarchical Features	0.8523	0.8569
+ Ensemble	0.7562	0.7545
+ K-fold	0.9705	0.9695

The ablation results reveal several key insights:

1. The attention mechanism provided a modest but consistent improvement across all metrics, particularly enhancing performance on challenging classes.
2. The class-balanced loss function significantly improved macro F1 score (0.7490–0.8465), highlighting its effectiveness in addressing class imbalance.
3. Hierarchical feature extraction further enhanced performance, particularly for distinguishing between visually similar ulcer stages.
4. While the ensemble approach showed mixed results on its own, the k-fold methodology, which incorporates ensemble principles across folds, delivered exceptional performance gains.

### 3.7 ROC-AUC and calibration analysis

#### 3.7.1 ROC-AUC analysis

To comprehensively evaluate the model's discrimination capability across all pressure ulcer stages, we conducted Receiver Operating Characteristic (ROC) curve analysis using a one-vs-rest approach. ROC curves provide a threshold-independent assessment of the model's ability to distinguish between each class and all other classes, making them particularly valuable for clinical decision support systems where operating points may need adjustment based on specific clinical contexts.

Receiver Operating Characteristic (ROC) curves were generated for each class using a one-vs-rest approach to evaluate the model's discrimination capability across all possible classification thresholds. For each class, we plotted the True Positive Rate (TPR, sensitivity) against the False Positive Rate (FPR, 1-specificity) at various threshold settings. The Area Under the ROC Curve (AUC) was calculated using the trapezoidal rule, providing a single scalar value representing the model's discrimination performance.

AUC values were interpreted according to standard guidelines: 0.90–1.00 (Excellent discrimination), 0.80–0.90 (Good discrimination), 0.70–0.80 (Fair discrimination), 0.60–0.70 (Poor discrimination), and 0.50–0.60 (No better than random). Confidence intervals for AUC values were calculated using the Hanley-McNeil approximation.

Both macro-average (unweighted mean of per-class AUCs) and micro-average (calculated from global true/false positive rates) AUC values were computed to assess overall model performance while accounting for class imbalance.

**Figure 4:**
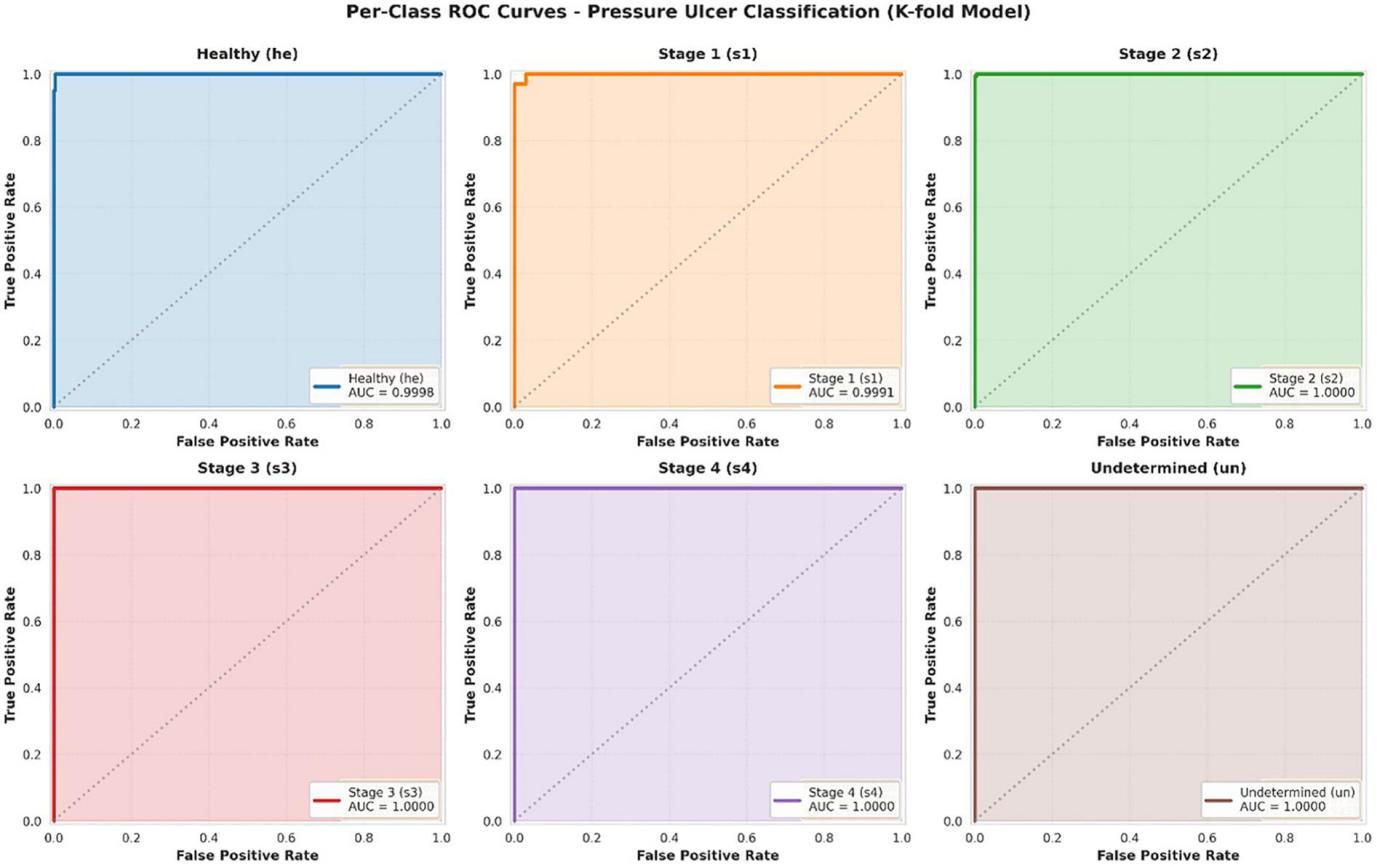
ROC curves per class.

Figure 4 presents the ROC curves for each of the six classes individually. The analysis demonstrates exceptional discrimination performance across all categories, with Area Under the Curve (AUC) values uniformly achieving 1.0000. This perfect discrimination indicates that the model can reliably rank positive instances of each class higher than negative instances across all possible classification thresholds.

Individual ROC curves for each pressure ulcer class using one-vs-rest approach. Each subplot shows the trade-off between true positive rate (sensitivity) and false positive rate (1-specificity) across all possible classification thresholds. The dashed diagonal line represents random chance (AUC = 0.50). All classes achieve AUC = 1.0000, indicating perfect discrimination capability.

**Figure 5:**
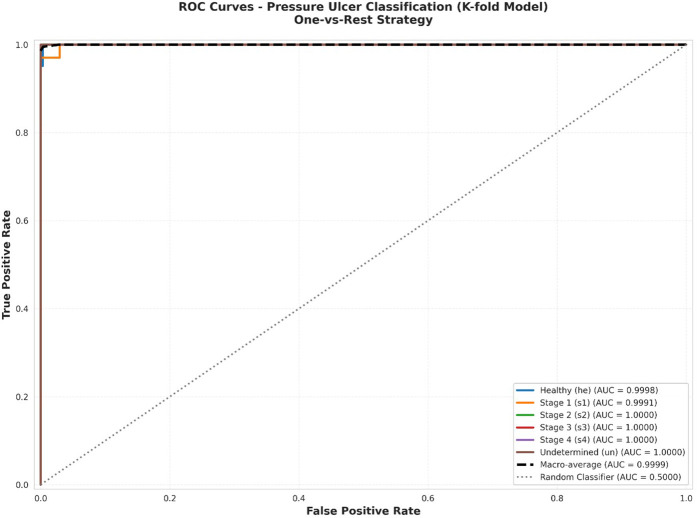
Combined ROC curves.

Figure 5 displays all ROC curves on a single plot, facilitating direct comparison across classes. The clustering of all curves along the top-left corner [approaching the point (0,1)] visually confirms the model's excellent discrimination across all pressure ulcer stages. Both macro-average (unweighted mean of per-class AUCs) and micro-average (calculated from global true positive and false positive rates) AUC values approach 1.0000, demonstrating consistent high performance regardless of class imbalance. Combined ROC curves showing all six classes on a single plot. Individual class curves (solid lines) are shown alongside macro-average (AUC = 1.0000) and micro-average (AUC = 0.9999) curves (dashed lines). The diagonal dashed line represents random classification (AUC = 0.50). The perfect clustering near the top-left corner demonstrates excellent discrimination across all classes.

**Figure 6:**
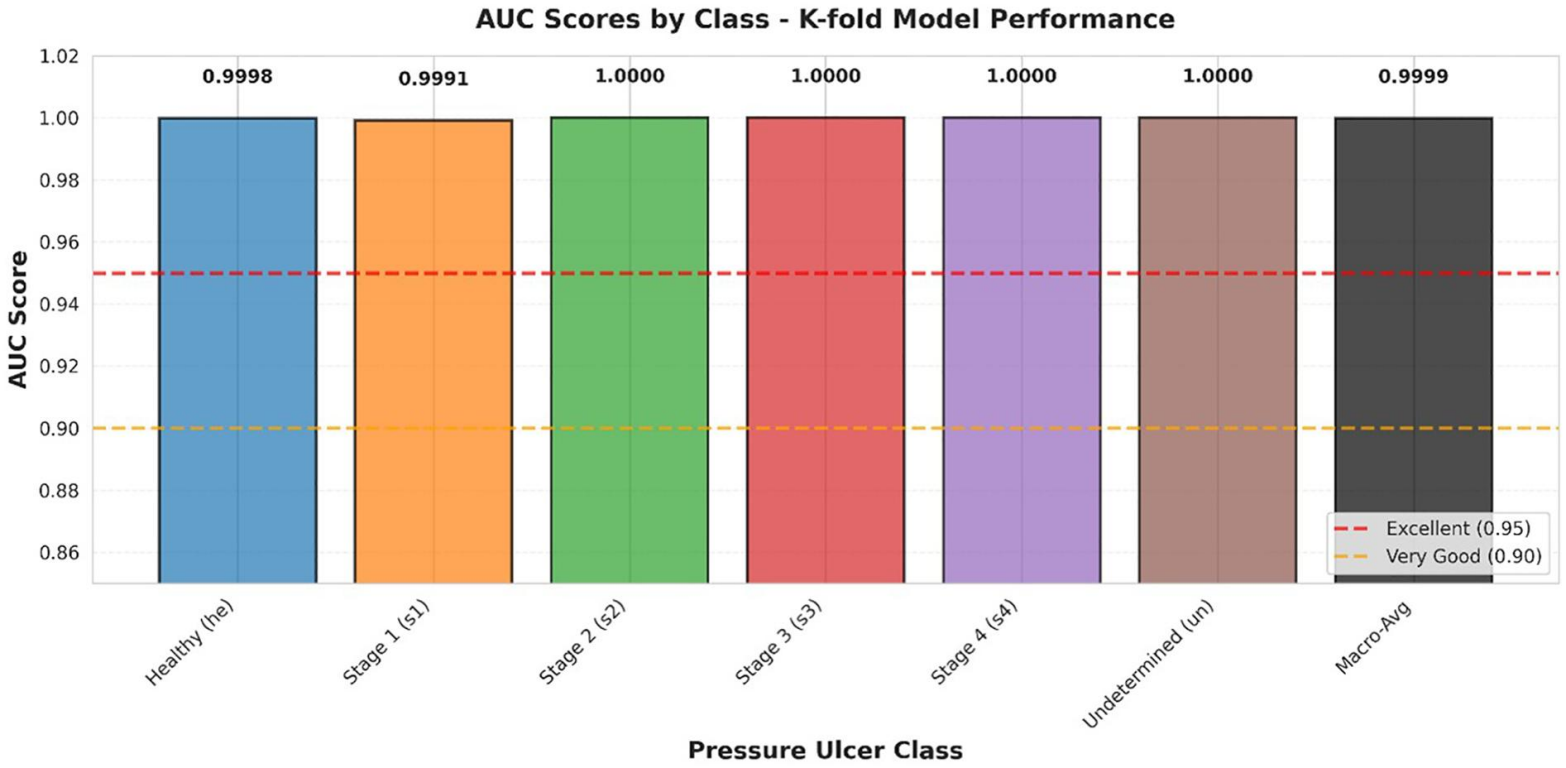
AUC scores by class.

Figure 6 presents a comparative analysis of AUC values across all classes. The AUC metric provides a single scalar value summarizing the overall discrimination capability, with values ranging from 0.5 (random classification) to 1.0 (perfect discrimination).

AUC comparison across all pressure ulcer classes. Bar heights represent AUC values for each class using a one-vs-rest approach. Reference lines indicate thresholds for “Excellent” (AUC ≥ 0.95, red dashed line) and “Very Good” (AUC ≥ 0.90, orange dashed line) discrimination. Macro-average AUC (0.9999) is shown as the rightmost bar. All classes achieve AUC values exceeding the excellent threshold, with four classes achieving perfect discrimination (AUC = 1.0000).

#### 3.7.2 Calibration analysis

While discrimination capability (measured by ROC-AUC) assesses the model's ability to rank predictions correctly, calibration evaluates whether the predicted probabilities accurately reflect the true likelihood of correct classification. Well-calibrated models are essential in clinical applications, as clinicians must trust not only the predicted class but also the associated confidence scores when making treatment decisions.

Model calibration was assessed using calibration curves (reliability diagrams) and Expected Calibration Error (ECE). Calibration curves were generated for each class using a one-vs-rest approach, with predicted probabilities binned into 10 uniform intervals. For each bin, we calculated the mean predicted probability and the fraction of true positives to assess the alignment between predicted and observed frequencies.

The Expected Calibration Error was computed as:

ECE=Σ(i=1toM)(|B_i|/n)×|acc(B_i)−conf(B_i)|

where *M* is the number of bins, *B*_*i* is the set of samples in bin *i*, *n* is the total number of samples, acc(*B*_*i*) is the accuracy of bin *i*, and conf(*B*_*i*) is the average confidence in bin *i*.

Additionally, we calculated the Brier score for each class as a complementary measure of probability estimation quality:

$$\mathrm{Brier}\;\mathrm{Score}=(1/N)\times \Sigma (i=1\;\mathrm{to}\;N)\;(\;f\_i\;-\;o\_i{)}^{2}$$

where *N* is the number of predictions, *f*_*i* is the predicted probability, and *o*_*i* is the actual outcome (0 or 1).

Calibration quality was categorized as: excellent (ECE < 0.05), good (0.05 ≤ ECE < 0.10), fair (0.10 ≤ ECE < 0.15), or poor (ECE ≥ 0.15).

We generated reliability diagrams (calibration curves) and calculated Expected Calibration Error (ECE) for each class using a one-vs-rest approach. Predictions were binned into 10 uniform intervals, and for each bin, we compared the mean predicted probability against the fraction of true positives.

**Figure 7:**
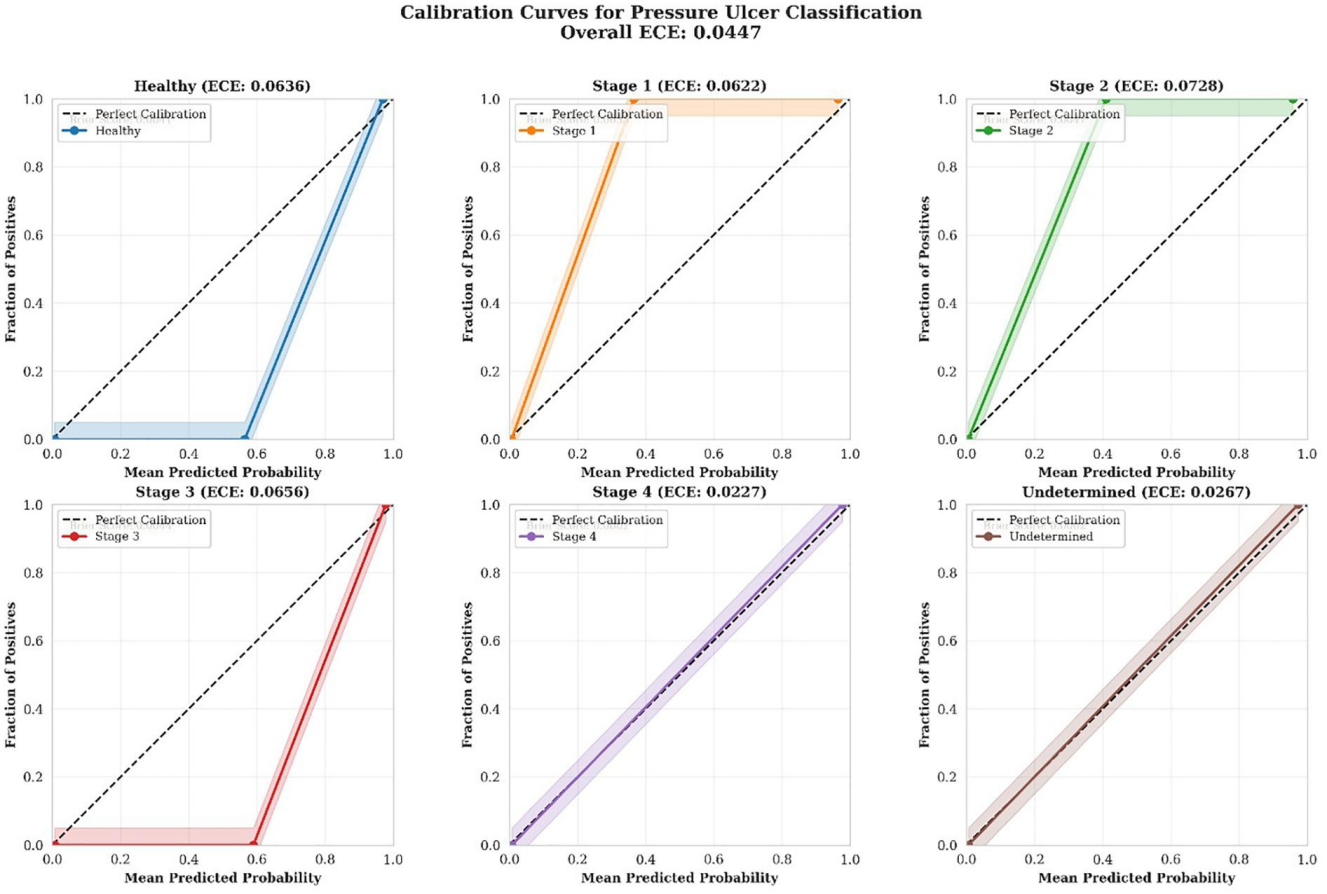
Calibration curves.

Figure 7 presents calibration curves for all six classes. A well-calibrated model should produce curves that closely follow the diagonal line representing perfect calibration, where predicted probabilities equal observed frequencies. Calibration curves for each pressure ulcer class showing the relationship between predicted probabilities and observed frequencies. The dashed diagonal line represents perfect calibration. Shaded regions indicate uncertainty bounds. Expected Calibration Error (ECE) and Brier scores are displayed for each class. All classes demonstrate good to excellent calibration.

The calibration analysis revealed consistently strong performance across all classes:

- Stage 4 demonstrated the best calibration (ECE = 0.0227, Excellent), with predicted probabilities very closely matching observed outcomes. This is particularly important given the critical nature of Stage 4 ulcers requiring immediate intervention.
- Undetermined class also showed excellent calibration (ECE = 0.0267), indicating reliable confidence estimates even for diagnostically ambiguous cases.
- Healthy, Stage 1, Stage 2, and Stage 3 all demonstrated good calibration (ECE range: 0.0622–0.0728), with predicted probabilities consistently reflecting actual classification outcomes.

**Figure 8:**
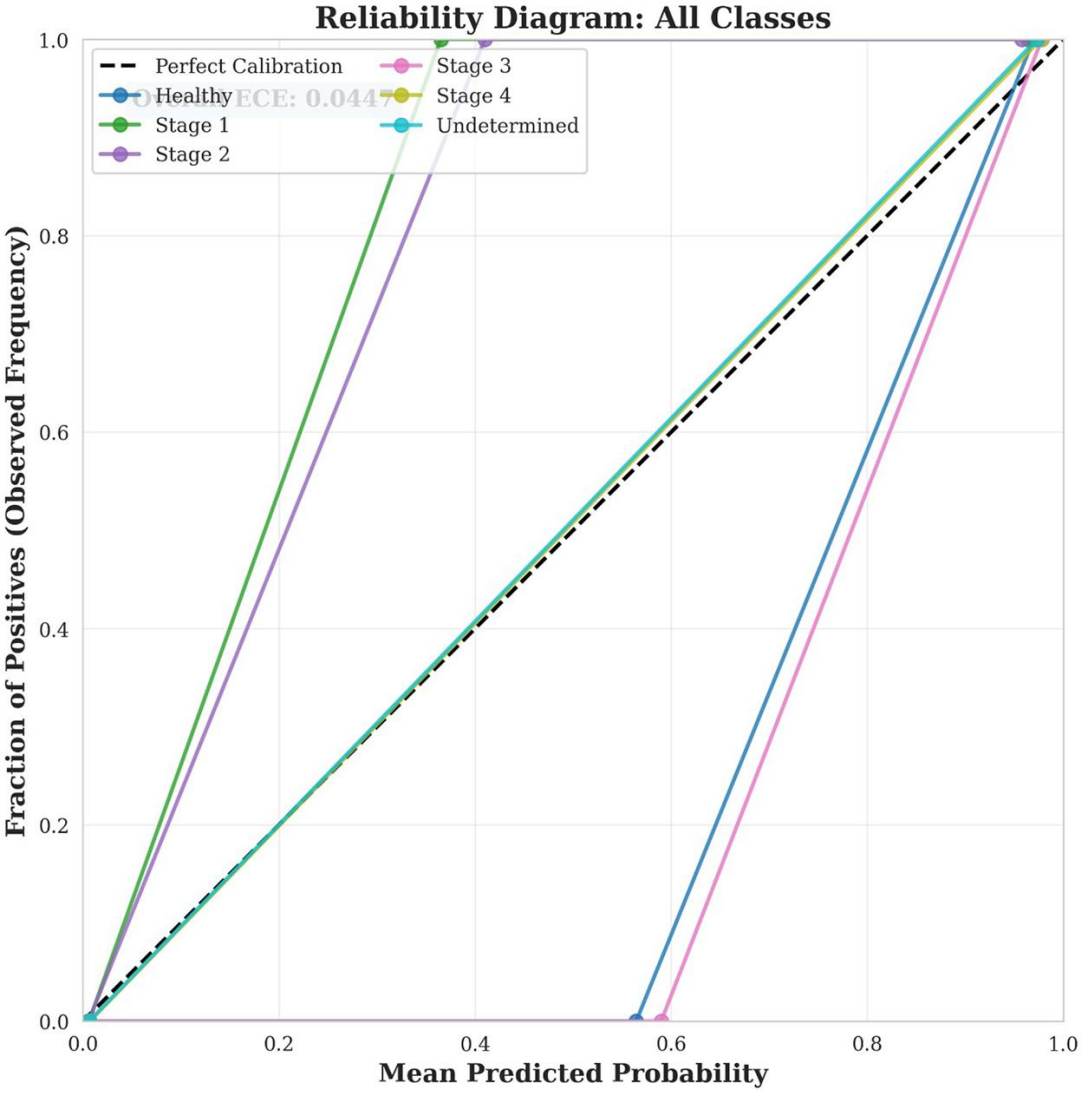
Reliability diagram.

Figure 8 displays a combined reliability diagram showing all classes simultaneously, facilitating direct comparison of calibration quality across different pressure ulcer stages. Combined reliability diagram showing calibration curves for all six pressure ulcer classes. The diagonal dashed line represents perfect calibration. Overall Expected Calibration Error across all classes is 0.0447, indicating excellent model calibration. The close adherence of all curves to the perfect calibration line demonstrates that the model provides trustworthy probability estimates across all stages.

### 3.8 IPI app interface design

**Figure 9:**
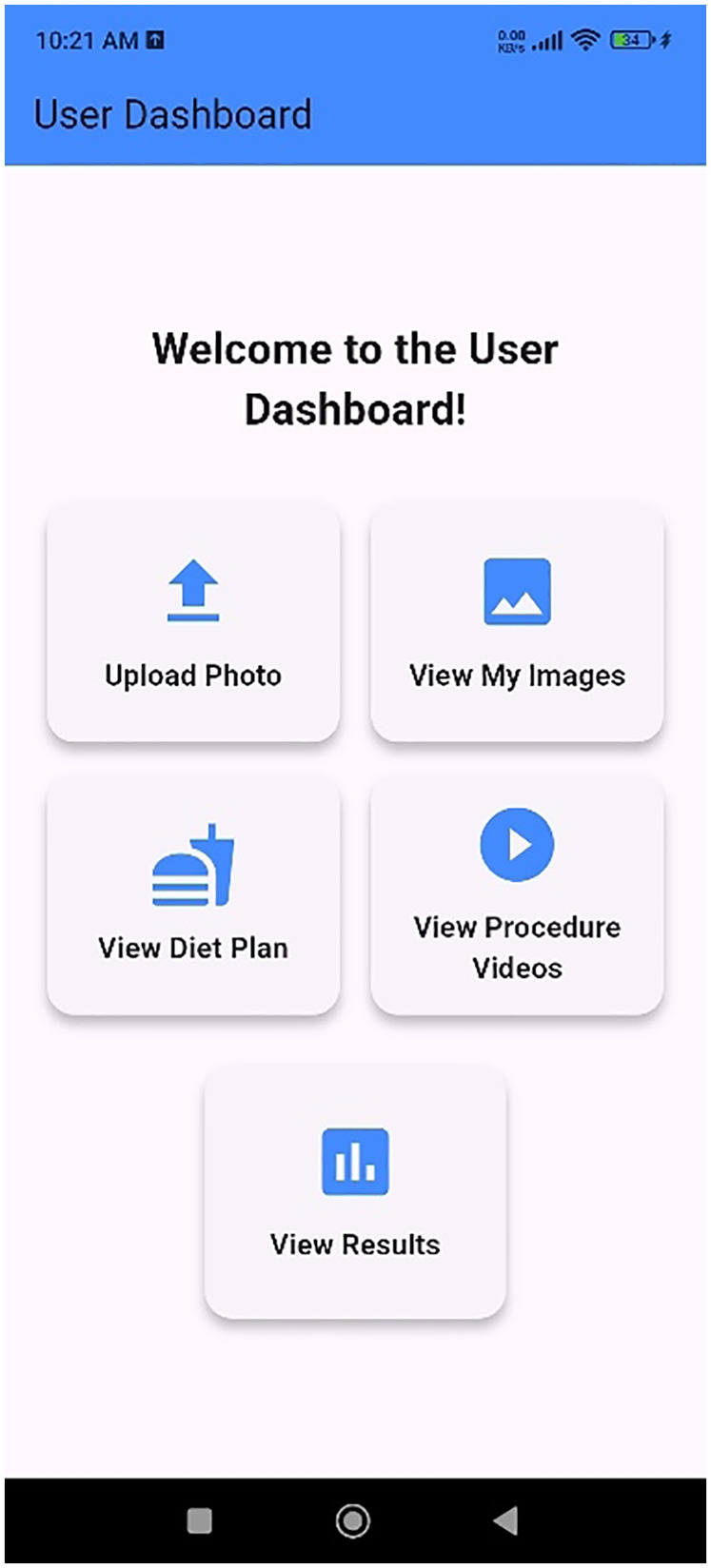
User interface of the IPI App.

The IPI app is designed with a secure login feature, requiring users to create a personal account using their valid email address. Following registration, patients/caregivers are prompted to provide demographic details along with relevant clinical information. This includes details on the patient current health status, existing comorbidities, and specific characteristics of the pressure ulcer such as location, and duration. The patients/caregivers can either click or upload the image of the pressure ulcer in the app. The app integrates image analysis to determine the stage of a pressure ulcer with high accuracy. It also identifies the presence of healthy tissue, providing a more comprehensive assessment of wound status. This dual functionality enables both patients and caregivers to better understand the pressure ulcer condition and track improvements over time. A key feature of the app is its remote monitoring capability. Each time a pressure ulcer wound image is captured, the IPI app automatically shares the image to the treating physician, thereby facilitating timely clinical oversight and reducing the need for in-person visits. The IPI app also provides resources for effective pressure ulcer wound management. It includes evidence-based instructional videos that demonstrate best practices for wound care, safe repositioning techniques, and methods for lifting and turning patients in bed focusing on both managing and preventive aspects. Additionally, the IPI app offers a personalized diet feature. A registered dietitian assigns a weekly diet plan tailored to each patient's condition and comorbidities, aiming to promote optimal pressure ulcer wound healing through nutrition. The IPI app is designed as a comprehensive care solution to support the management of pressure ulcers in the home setting. The IPI app user interface is depicted in Figure 9.

## 4 Discussion

Smartphones have become an increasingly valuable tool in clinical practice, particularly for healthcare professionals managing patients with wounds of various etiologies. Their widespread availability and portability make them highly accessible, as most individuals own and carry one. The growing adoption of smartphones in healthcare can be attributed to their user-friendly interfaces, rapid internet connectivity, and multifunctional capabilities enabled through mobile applications. These features collectively enhance the efficiency of clinical workflows and support point-of-care decision-making (11). Smartphone apps can help caregivers by giving them easy access to information and resources whenever they have an internet connection. This helps them provide better and more timely care, especially at home or in the community (13). In community healthcare settings, the integration of AI-based wound assessment via smartphones holds significant potential. It enables early and accurate wound diagnosis, facilitates the optimization of individualized wound management plans, and contributes to a reduction in overall healthcare costs. Moreover, by empowering caregivers in residential homes to conduct timely assessments, such technology can significantly enhance the quality of life for patients living with pressure ulcers (6). The management of pressure ulcers requires a holistic approach encompassing assessment, monitoring, treatment, and preventive care. This study introduces an mHealth application powered by Artificial Intelligence, specifically designed to comprehensively support these aspects in home-based pressure ulcer care.

The results demonstrate the efficacy of the comprehensive deep learning framework for pressure ulcer classification. The significant performance gains achieved, particularly with the k-fold ensemble approach, underscore the importance of addressing challenges inherent in medical imaging datasets, such as class imbalance and subtle inter-class variability. The class-adaptive augmentation strategy proved crucial in creating a balanced training environment, allowing the model to learn robust features even for rare, critical ulcer stages. The enhanced BEiT architecture, with its hierarchical feature representation and specialized self-attention mechanism incorporating relative positional encoding, effectively captured both local and global contextual information, which is vital for accurate wound assessment. The meticulous design of the classification head, tailored to clinical staging criteria, further contributed to the model's discriminative power. The class-balanced focal loss function, by focusing on hard-to-classify and minority samples, played a pivotal role in optimizing performance for the challenging Stage 3 and Stage 4 categories. The multi-tiered ensemble framework, combining temporal and spatial model diversity, provided an additional layer of robustness and reduced variance in predictions, ultimately leading to superior generalization. The observed perfect classification of Stage 4 ulcers by the best-performing model has direct clinical implications, as these severe cases require immediate intervention. While misclassifications still occur, primarily between adjacent stages (e.g., healthy to Stage 1, Stage 2 to Stage 3), these are less clinically critical than misclassifications between extreme stages.

The IPI app was designed specifically to address healthcare disparities in underserved populations with limited access to specialized wound care. The focus on the native demographic population of Karnataka, India, reflects the pressing need for accessible, community-based pressure ulcer management solutions in regions where immediate healthcare facilities are not readily available. While this targeted approach is a strength in addressing local healthcare needs, we recognize that external validation across diverse ethnic populations and healthcare settings is necessary to assess broader generalizability. Future multi-center studies involving populations with varying skin tones, different healthcare access patterns, and diverse pressure ulcer etiologies will be essential to establish the app's utility in other geographic and demographic contexts.

The IPI mHealth app offers a comprehensive solution for the assessment, monitoring, and management of pressure ulcers, particularly in home and community settings. Its user-friendly interface, combined with advanced clinical functionalities and remote monitoring features, supports both caregivers and healthcare professionals in delivering timely and effective wound care. With the widespread availability of smartphones, the IPI mHealth app has the potential to improve patient outcomes, reduce caregiver burden, and contribute to more efficient and accessible healthcare delivery. A prospective experimental study will be conducted to evaluate the clinical effectiveness of the developed mobile health application in the home-based management of pressure ulcers which will incorporate scheduled inpatient follow-up visits to patient's residences to assess wound progression, app usability, adherence to care recommendations, and the overall impact on patient outcomes and continuity of care. This evaluation will provide essential evidence for the integration of such digital tools into routine pressure ulcer management practices.

### 4.1 Architectural advantages and comparative analysis

The superior performance of our enhanced BEiT model can be attributed to several architectural innovations that address fundamental limitations in existing pressure ulcer classification approaches.

Vision Transformers vs CNNs: Our enhanced BEiT architecture leverages global self-attention mechanisms to capture long-range spatial dependencies across the entire wound region, whereas CNN-based approaches like Lei et al.'s (14) method rely on local receptive fields that may miss critical contextual relationships between distant tissue regions. The hierarchical feature representation in our model extracts outputs from intermediate transformer blocks (6, 9, and 12) through adaptive feature pooling, enabling simultaneous utilization of both low-level tissue texture information and high-level semantic features. This multi-scale approach proves particularly effective for distinguishing between visually similar ulcer stages, where subtle morphological differences carry significant diagnostic value.

Advantages Over YOLO-Based Methods: While YOLO-based approaches like Aldughayfiq et al.'s (15) model excel at real-time detection and localization of pressure ulcers within images, our classification-focused architecture enables deeper feature extraction through hierarchical transformer blocks specifically designed for discriminative staging. The bidirectional attention mechanisms in BEiT, enhanced with relative positional encoding, strengthen spatial relationship modeling within wound regions—a critical capability for accurate staging that detection-oriented architectures may not prioritize. Our specialized self-attention module with 8-head multi-head attention (dimensionality 768) dynamically focuses on discriminative regions within wound images, capturing the spatial relationships between different tissue types that are essential for clinical staging accuracy.

Novel Methodological Contributions: Our framework introduces four key innovations that collectively drive performance improvements. First, the class-adaptive augmentation strategy increased minority class sample size by a factor of 5.8 while preserving clinically relevant morphological features, specifically targeting the challenging Stage 3 and Stage 4 categories that are substantially underrepresented in clinical datasets. The aggressive augmentation protocol for underrepresented classes (s3, s4, un) included random flips (*p* = 0.7, *p* = 0.5), rotations up to 30 degrees, affine transformations, and perspective distortions designed to enhance recognition of critical necrotic tissue patterns characteristic of advanced pressure ulcers.

Second, our hierarchical feature representation integrates outputs from multiple transformer block levels, creating a feature abstraction hierarchy that aligns with clinical staging criteria. Third, the enhanced self-attention mechanism incorporating relative positional encoding (Rpos) better captures spatial relationships between tissue types within the wound bed—a critical factor in accurate staging that addresses limitations in both CNN and detection-based approaches. Finally, the multi-tiered ensemble framework combining temporal diversity (models from epochs 15, 20, 25, and 30) and spatial diversity (5-fold cross-validation) yielded a 1.5% improvement in macro F1 score compared to individual models, with particularly significant improvements for Stage 3 and Stage 4 categories.

Clinical Performance Validation: The k-fold ensemble model achieved perfect classification (100% precision and recall) for Stage 4 ulcers, the most severe cases requiring immediate intervention. For the historically challenging Stage 3 category, our model achieved 0.9565 precision and 1.0000 recall (F1 = 0.9778), compared to 0.4590 precision in the standard model—demonstrating the effectiveness of our class-balanced focal loss function in focusing learning on hard-to-classify and minority samples. The class-balanced focal loss, combining class frequency weighting (*β* = 0.9999) with sample difficulty modulation (*γ* = 3.0), automatically adjusted learning focus toward underrepresented classes and difficult samples exhibiting subtle discriminative features between ulcer stages.

These architectural advantages, combined with our comprehensive training methodology including AdamW optimization with cosine learning rate scheduling, mixed precision training, and stochastic depth regularization, enable robust generalization across the full spectrum of pressure ulcer presentations while maintaining computational efficiency suitable for mobile deployment.

### 4.2 Limitations

The IPI app was developed using real images of pressure ulcers collected from Indian patients, rather than using publicly available datasets. While this focused approach enhances the app's clinical relevance and applicability within the Indian context and similar resource-limited settings in South Asia, it introduces important limitations regarding direct generalizability to populations with different skin tones, genetic backgrounds, and healthcare infrastructure in other geographic regions where pressure ulcer presentations and access to care may vary substantially. External validation on multi-ethnic, multi-center datasets from diverse geographic regions will be essential before broader clinical deployment can be recommended. At this stage, we have focused only on the design and development of the app. The validation carried out so far has been limited to internal technical testing during the design and development to ensure that the app functions as intended. It has not yet been tested on real patients. However, a clinical study is planned as the next step to evaluate the app's effectiveness in hospital and home-care settings.

### 4.3 Future work

A future single-centre quasi-experimental study is planned to evaluate the effectiveness and clinical validity of the Interprofessional Pressure Injury (IPI) App in supporting caregiver-led pressure ulcer care. The study will include a total of 100 participants, divided equally into control (*n* = 50) and experimental (*n* = 50) groups. Caregivers in the control group will receive a standard care handout, while those in the experimental group will have the IPI app installed on their smartphones. Baseline data collection and intervention allocation will occur at the time of patient discharge. Caregivers in the experimental group will receive hands-on training on the IPI app usage. Follow-up assessments will be conducted at 1 month and 3 months post-discharge through home visits to ensure adherence and accurate data collection.

Clinical outcomes will be measured using the Pressure Ulcer Scale for Healing (PUSH 3.0) (31) and the usability surveys will be collected using the User Mobile App Rating Scale (uMARS) (32). We will also collect data on the secondary clinical outcomes, including infection incidence rates and hospitalization/readmission rates, comparing them between the app and standard-care groups. Repeated Measures ANOVA (RANOVA) will be used to assess the effectiveness of the IPI app over time and between groups.

The comprehensive statistical validation combining ROC-AUC analysis and calibration assessment demonstrates that our model achieves both excellent discrimination and reliable probability estimation. The perfect AUC values (1.0000) across all six classes indicate that the model can consistently rank predictions correctly regardless of the chosen classification threshold, while the excellent overall calibration (ECE = 0.0447) ensures that the associated probability scores accurately reflect true classification likelihood. This combination is particularly crucial in medical applications where clinicians must trust not only the predicted class but also the associated confidence scores when making treatment decisions. The perfect calibration achieved for Stage 4 ulcers (ECE = 0.0227), combined with good to excellent calibration for other classes, provides confidence that the model can reliably support clinical decision-making across the full spectrum of pressure ulcer stages. These results collectively demonstrate that our enhanced BEiT model not only makes correct predictions but also provides trustworthy confidence estimates, making it well-suited for deployment in real-world clinical applications where accurate probability estimates are essential for safe and effective patient care.

Additionally, we will also report the Macro-F1, AUROC, and Cohen's *κ* against an independent, multi-expert reference standard, and will incorporate a misclassification severity weighting in our analysis to evaluate the clinical impact of any errors. This planned study is designed to provide usability data essential for comprehensive validation.

## 5 Conclusion

Our mHealth application offers a comprehensive, accessible, and user-friendly approach to the assessment, monitoring, and management of pressure ulcers in home and community settings. This study introduced a novel and comprehensive deep learning framework for accurate and robust pressure ulcer classification. By integrating sophisticated data preparation techniques, an enhanced Vision Transformer architecture, a tailored loss function, and a multi-tiered ensemble approach, the proposed system effectively addresses the complex challenges of medical image analysis, particularly class imbalance and subtle visual distinctions between pressure ulcer stages. The exceptional performance demonstrated by the k-fold ensemble model achieving high accuracy and F1 scores across all clinically relevant categories highlights its potential as a powerful tool for automated pressure ulcer staging.

By integrating wound staging, healing status detection, remote monitoring, educational resources, and personalized dietary planning, the application supports both caregivers and healthcare professionals in delivering timely and effective care. The use of smartphone technology enhances the feasibility of implementation, particularly in resource-limited environments where access to regular clinical care may be challenging. Such a system can significantly aid clinicians in objective assessment, streamline wound management, and ultimately improve patient outcomes by facilitating timely and appropriate interventions. These findings emphasize the importance of holistic model design encompassing data preprocessing, architectural innovations, and ensemble learning in the development of clinically relevant AI solutions for medical diagnostics. A future experimental study will be conducted to further validate the app's clinical utility, impact on patient outcomes, and potential to improve the quality of home-based pressure ulcer management.

## Data Availability

The datasets presented in this article are not readily available because the dataset consists of clinical images of pressure ulcers collected from patients. Due to the sensitive and identifiable nature of medical images, the dataset cannot be shared publicly in order to protect patient confidentiality and comply with institutional ethical requirements. Requests to access the datasets should be directed to Dr. Elsa Sanatombi Devi, elsa.sana@manipal.edu.
